# Supplementary material for: Propagation of Mitochondria-Derived Reactive Oxygen Species within the Dipodascus magnusii Cells
Source: Antioxidants (Basel). 2021 Jan 15;10(1):120. doi: 10.3390/antiox10010120 (PMC7830518; doi:10.3390/antiox10010120)
Supplement: Supplementary file 1 [file antioxidants-10-00120-s001.zip › antioxidants-1040177-supplementary.pdf]

Supplementary materials

Video S1. Reactive oxygen species (ROS) production in *Dipodascus magnusii* cells measured by time-lapse fluorescence imaging. Cells harvested at the exponential growth phase were rinsed with 50 mM PBS, pH 5.5, loaded with 1 μM MitoSox Red and 15 μM H2DCF-DA for 30 min, then washed in a fresh portion of incubation medium. Images were obtained every 3min. Red fluorescence is specified for the oxidized MitoSox Red; green fluorescence—for DCF.

Video S2. Development of *tert*-butyl hydroperoxide (*t*-BHP)-induced reactive oxygen species (ROS) production in *Dipodascus magnusii* cells measured by time-lapse fluorescence imaging. Cells harvested at the exponential growth phase and rinsed with 50 mM PBS, pH 5.5, were loaded with 15 μM H_2DCF-DA and 1 μM MitoSOX Red for 30 min, then washed in a fresh portion of incubation medium and exposed to 750 μM *t*-BHP. Images were obtained every 3min. Red fluorescence is specified for the oxidized MitoSox Red; green fluorescence—for DCF.

Video S3. Effect of SkQ1 on the propagation *tert*-butyl hydroperoxide (*t*-BHP)-induced reactive oxygen species (ROS) in *Dipodascus magnusii* cells measured by time-lapse fluorescence imaging. Cells harvested at the exponential growth phase were preincubated with 800 nM SkQ1 for 1 h, rinsed with 50 mM PBS, pH 5.5, loaded with 1 μM MitoSox Red and 15 μM H2DCF-DA for 30 min, then washed in a fresh portion of incubation medium and exposed to 750 μM *t*-BHP.

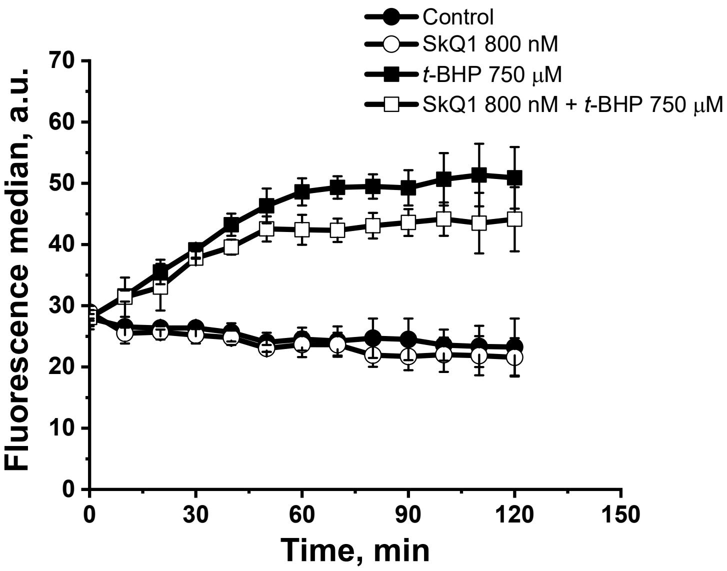

Figure S1. Lipid peroxidation in *Dipodascus magnusii* cells exposed to *tert*-butyl hydroperoxide (*t*-BHP) measured by flow cytometry. Effect of SkQ1. Cells harvested at the exponential growth phase and rinsed with 50 mM PBS, pH 5.5, were loaded with 200 nM MitoCLox for 30 min, then washed in a fresh portion of incubation medium and preincubated with 800 nM SkQ1 for 1 h, then washed in a fresh portion of incubation medium and exposed to 750 μM *t*-BHP. Cells were examined every 10 min.

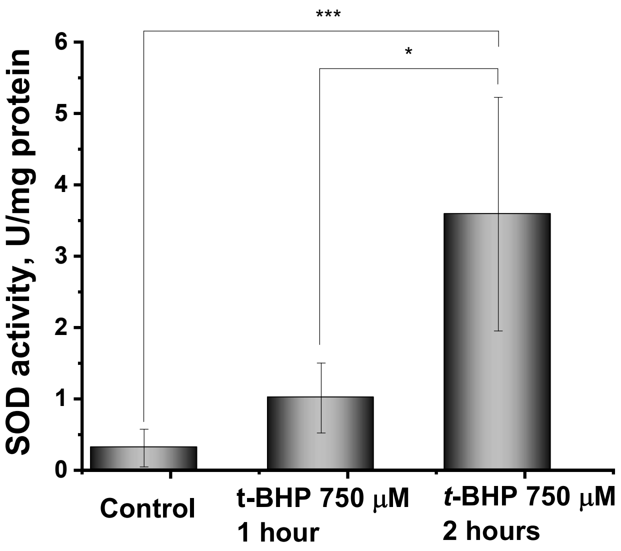

Figure S2. Superoxide dismutase (SOD) activity in *Dipodascus magnusii* cells exposed to 750 µM *t*-BHP. Cells harvested at the exponential growth phase were exposed to 750 µM *t*-BHP for 1 and 2 h, then washed with 50 mM PBS, pH 5.5, suspended in assay buffer, then disrupted and the supernatant was used for determination of enzymatic activity. The statistical analyses were carried out by the one-way ANOVA test. * reactive oxygen species ***: $0.001 < p < 0.01$; *: $0.01 < p < 0.05$.
